# Trends and Perspectives of Biological Drug Approvals by the FDA: A Review from 2015 to 2021

**DOI:** 10.3390/biomedicines10092325

**Published:** 2022-09-19

**Authors:** Alexander C. Martins, Mariana Y. Oshiro, Fernando Albericio, Beatriz G. de la Torre, Gustavo José V. Pereira, Rodrigo V. Gonzaga

**Affiliations:** 1 School of Health Sciences, UAM, Universidade Anhembi-Morumbi, São Paulo 03101-001, Brazil; 2 School of Chemistry and Physics, University of KwaZulu-Natal, Durban 4001, South Africa; 3 CIBER-BBN, Networking Centre on Bioengineering, Biomaterials and Nanomedicine, Department of Organic Chemistry, University of Barcelona, 08028 Barcelona, Spain; 4 KRISP, College of Health Sciences, University of KwaZulu-Natal, Durban 4001, South Africa; 5 FCF-USP, School of Pharmaceutical Sciences, University of Sao Paulo, Sao Paulo 05508-000, Brazil; 6 Centro Universitário São Camilo, São Paulo 04262-200, Brazil; 7 FAM—Centro Universitário das Américas, São Paulo 01304-001, Brazil

**Keywords:** Food and Drug Administration, FDA approvals, monoclonal antibody, antibody–drug conjugate, first global approval, biological drugs

## Abstract

Despite belonging to a relatively new class of pharmaceuticals, biological drugs have been used since the 1980s, when they brought about a breakthrough in the treatment of chronic diseases, especially cancer. They conquered a large space in the pipeline of the pharmaceutical industry and boosted the innovation portfolio and arsenal of therapeutic compounds available. Here, we report on biological drug approvals by the US Food and Drug Administration (FDA) from 2015 to 2021. The number of drugs included in this class grew over this period, totaling 90 approvals, with an average of 13 authorizations per year. This figure contrasts with previous periods, which registered between 2 and 8 approvals per year. We highlight the great potential and advantages of biological drugs. In this context, these therapeutics show high efficacy and high selectivity, and they have brought about a significant increase in patient survival and a reduction of adverse reactions. The development and production of biopharmaceuticals pose a major challenge because these processes require cutting-edge technology, thereby making the drugs very expensive. However, we believe that, in the near future, biological medicines will be more accessible and new drugs belonging to this class will become available as new technologies emerge. Such advances will enhance the production of these biopharmaceuticals, thereby making the process increasingly profitable and less expensive, thereby bringing about greater availability of these drugs.

## 1. Introduction

Biological drugs (or biopharmaceuticals) derive from living organisms. They are highly selective, high-cost, typically susceptible to microbial contamination, and generally temperature-sensitive drugs. They can also be used as advanced alternatives when conventional synthetic drugs no longer have the desired effect [1].

Biopharmaceuticals can be isolated from microorganisms, humans, animals or they can be isolated from compounds of nucleic acids, sugars and proteins. Here, we will address authorizations given by the U.S. Federal Drug Administration (FDA) to biologicals classified as monoclonal antibodies (mAbs), antibody–drug conjugates (ADCs), and proteins, which encompass enzymes and hormones [2]. All product references cited in this work hold a Biologics License Application (BLA) number. Although we will not include biosimilars in the quantitative analysis, they will be briefly commented on.

Advances in biological drug development by the pharmaceutical industry have given rise to new treatments to meet urgent medical needs, among them cancer. For example, regarding biologicals to treat diseases like cancer and autoimmune conditions, in 2014, four mAbs were indicated for cancer. More recently, in 2020, this figure had doubled, with eight mAbs for the treatment of this disease, while in 2021 there were five mAbs for this purpose. In the context of autoimmune diseases, in 2014, there was only one mAb and one enzyme approved, while in 2016 there were two mAbs, and in 2017 four [3,4]. It was only from 2015 onward that the number of approvals of biologicals per year jumped to a 2-digit figure as prior to 2015 such approvals did not reach 10 per year.

This retrospective observational review covers all the biologicals approved for the treatment of cancer, autoimmune conditions, and all other diseases, including rare diseases, between 2015 and 2021. It also encompasses many examples of the effectiveness of this class of drugs. We have excluded articles that mentioned the approvals of biologicals for COVID-19 and also articles about biosimilars. All the biosimilars mentioned briefly herein were found in the FDA Purple Book database.

## 2. Monoclonal Antibodies (mAbs)

The most common biologicals, mAbs are highly selective and they can be conjugated to chemical compounds, drugs, and toxins. They can be classified and named on the basis of their structure (Table 1), with the letter preceding the suffix -mab indicating the origin of the antibody. Murine mAbs comprise the constant and variable regions of the antibody from mice and they carry the suffix -omab in the name (Muronomab C3). Chimeric mAbs are composed of murine variable regions fused onto human constant regions, and they carry the suffix -ximab (Infliximab). Humanized mAbs are formed almost entirely by human regions of the antibody, except the complementary region, which is the antigen-binding region. This class of mAbs has the suffix -zumab (Galcanezumab). Therefore, Chimeric mAbs are more non-human based than Humanized mAbs. Fully human antibodies have the suffix -umab in their name (Adalimumab). In 1992, Muronomab C3 Orthoclone-OKT3^TM^, a murine mAb, was the first of such molecules to be licensed for commercialization. Studies revealed that these antibodies presented risks of patient-related immunogenicity. The immunogenicity of murine mAbs was found to be related to immune activation, which can be critical to patients, and this drove the need to evaluate this immunological trigger [5]. To tackle this issue, other classes of mAbs were developed years later [6,7]. Of note, the nomenclature of biopharmaceuticals is not limited to what is described in this work, and it is possible to find biologicals identified in a different manner in the literature (Table 1).

## 3. Antibody–Drug Conjugate (ADC)

**Figure 1:**
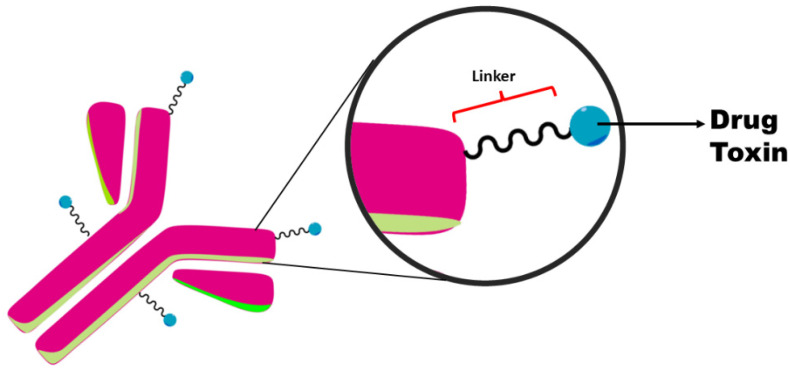
Schematic of the structure of an antibody–drug conjugate (ADC).

One of the main components of the ADC structure is also a mAb, which serves to increase selectivity by targeting the drug to tumors. ADCs have three main components, namely the mAb, a drug or toxin, and a biodegradable linker. The latter are characterized by being acid-cleavable, protease-cleavable, and hydrolysis-cleavable, or they can be cleaved by lysosomal enzymes before being released at the target [8] (Figure 1). These conjugated drugs have high selectivity, and they limit the exposure of healthy tissues to the cytotoxic agent. By providing the link between antibody and drug, the chemical ligand has the characteristic of being stable so that the drug can travel through the body and be released into the target tissues. In addition to showing stability in physiological conditions, the cytotoxic payload must have a conjugation functional group that allows it to be released in the desired manner to the target. [9,10]. For example, Moxetumomab Pasudotox Lumoxiti^TM^, which is used to treat capillary leukemia, is the conjugate of a fragment of an anti-CD22 mAb with a fragment of *Pseudomonas exotoxin* [11]. Therefore, this ADC combines the tumor-targeting capacity of the antibody and the antitumor activity of the toxic payload.

## 4. Proteins

This class also includes enzymes, growth hormone, IgG blockers, and also human interleukins. An example of a member of this group is Tagraxofusp Elzonris^TM^, an interleukin-3 (IL-3) with a payload of a truncated diphtheria toxin used to treat blastic plasmacytoid dendritic cell neoplasm (BPDCN) in adult and pediatric patients [12]. Tagraxofusp Elzonris^TM^ binds to CD123 and then the cytotoxic diphtheria toxin is released. 

Proteins can also be identified by their names. In this regard, they carry a different suffix, with -fusp for fusion proteins (for example, the previous one mentioned Tagraxofusp [13]) and -ase for enzymes, such as Calaspargase pegol Asparlas^TM^ used for the treatment of acute lymphoblastic leukemia. When the name of a protein is accompanied by the word pegol, it indicates pegylation of the molecule [7]. 

## 5. Biosimilars and Interchangeability

To shed light on the FDA approval of biosimilars, let us take as an example the biological reference Adalimumab Humira^TM^, the first antibody of fully human origin, which was approved in 2002 [7]. Between 2016 and 2021, the FDA database lists seven other Adalimumab drugs as biosimilars (Adalimumab-fkjp Hulio^TM^, Adalimumab-adaz Hyrimoz^TM^, Adalimumab-aqvh Yusimry^TM^, Adalimumab-bwwd Hadlima^TM^, Adalimumab-atto Amjevita^TM^, Adalimumab-afzb Abrilada^TM^, Adalimumab-adbm Cyltezo^TM^), only one of them (Cyltezo^TM^) being interchangeable with Humira^TM^. This high number of biosimilars may be explained by the fact that the authorization process is not as expensive as for reference biopharmaceuticals and there are patent rights involved, once the reference biological drug loses its right patent, a biosimilar can be developed. Manufacturers of a proposed biosimilar are not exempt from testing and must submit data proving that the drug is highly similar to the reference biopharmaceutical in structure, safety, and purity, including immunogenicity, pharmacokinetics and/or pharmacodynamics assessments. In other words, a shorter path to achieving the approval of a biosimilar does not imply a less reliable process [14]. 

## 6. Timeline for FDA-Approved Biological Drugs

**Figure 2:**
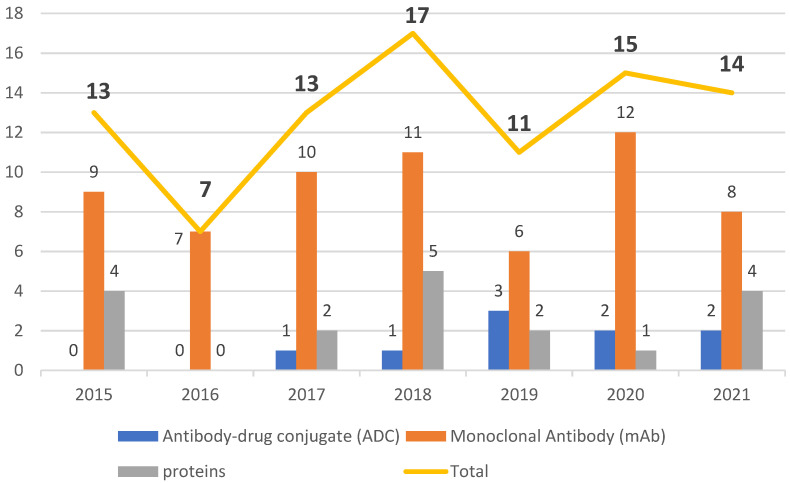
Biologicals approved by the Food and Drug Administration (FDA) from 2015 to 2021 [2,15,16,17,18,19,20,21].

The data collected in the present work point to an undeniable growth of biological therapies. In the period from 2015 to 2021, the FDA authorized new mAbs, ADCs and proteins. Of note, the total number of approvals remained in double figures every year except 2016, in which only seven biopharmaceuticals, all mAbs, were approved (Figure 2). Analysis of the data also revealed the prominence of the authorization of mAbs compared to other biologicals.

Although this work collects data from 2015 onwards, the last two years of the period of interest (2020 and 2021) were remarkable for several reasons. First, the COVID-19 pandemic promoted emergency use authorization for synthetics and biologicals, and second, the green light was given for the first biological for Alzheimer’s Disease (AD), namely Aducanumab Aduhelm^TM^. The approval of this drug met with criticism for its cost (US$56,000/year, with a reduction to US$28,200/year after a few months) and the manner that it received such authorization [22,23,24]. Moreover, it has been reported that Aducanumab may be related to severe adverse events such as brain swelling [25]. In this regard, the European Medicines Agency (EMA) and the Pharmaceuticals and Medical Devices Agency (PMDA) withdrew marketing authorization for this drug in 2021 [26]. AD is an extremely important medical target as, according to the FDA, there has not been a new treatment for this disease since 2003 [15].

**Figure 3:**
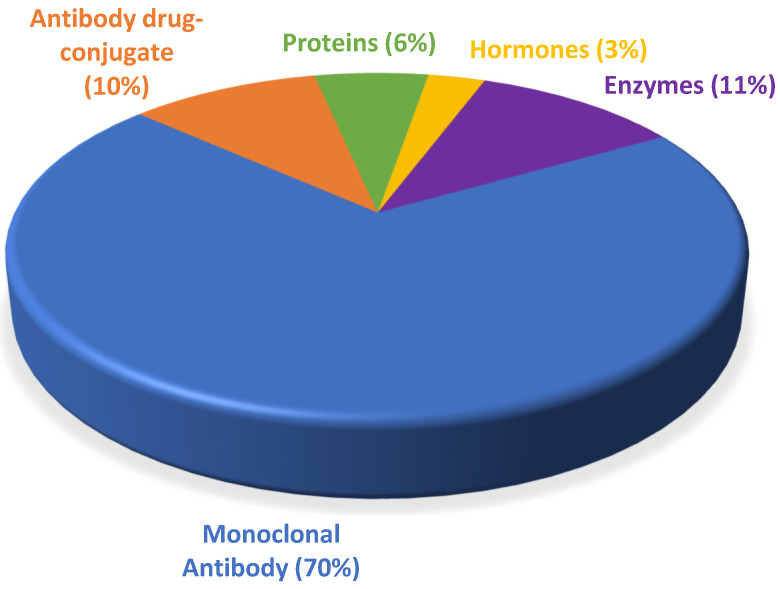
Percentage of new biopharmaceuticals approved by the Food and Drug Administration (U.S. FDA) from 2015 to 2021 [2,17,18,19,20,21].

The number of mAbs authorized each year between 2015 and 2021 has never been below 50% of total approvals (2015, 69.2%; 2016, 100%; 2017, 76.9%; 2018, 64.7%; 2019, 53%; 2020, 80%; and 2021, 57.1%). The next category of drugs in terms of the number of approvals in this period is enzymes (11%), followed by ADCs (10%), proteins and fusion proteins (6%), and finally hormones (3%) (Figure 3).

## 7. Therapeutic Indications

### 7.1. Cancer

Considering all the therapeutic targets found, the approvals of biopharmaceuticals for the treatment of cancer are highlighted in the period 2015–2021. A total of 32 biologicals were authorized for the treatment of a variety of cancers (cervical cancer, lymphomas, leukemias, and other blood cancers, lung cancer, endothelial cancer, sarcomas, carcinomas, breast cancer, multiple myeloma, neuroblastoma, skin cell cancers, among others). Of these, 62.5% (20) are mAbs, 30% (9) ADCs, and 9.37% (3) fall into the class of proteins (proteins and enzymes). Of note, the biologicals for the treatment of different types of cancer varied greatly from year to year, although mAbs were on the rise. In this context, in 2015, four out of the nine approved mAbs were for cancer, and in 2016 all six mAbs were for this indication. In 2017, of the three biopharmaceuticals for cancer, two were mAbs and one was an ADC. In 2018, of the five approvals for cancer, only two were mAbs, one was an ADC, and two were proteins. In 2019, of the four approvals for this indication, only one was a mAb, while the remaining three were ADCs. In 2020, six mAbs and two ADCs were authorized for the treatment of this disease. In 2021, only one mAb was approved, while two ADCs and one enzyme received the green light. The coronavirus over the last two years may have influenced the FDA’s decisions regarding the approval of new drugs, whether synthetic products or biologicals. 

### 7.2. Mechanisms of Action and Therapeutic Indications of ADCs and mAbs for Cancer

#### 7.2.1. mAbs for Cancer

Both IgG1k Daratumumab Darzalex^TM^ and the IgG1 Isatuximab Sarclisa^TM^ bind to CD38 [27,28]. Like other conventional medicines, biologicals can undergo changes. One example is Darzalex^TM^ (given intravenously), which was modified and approved in 2020 as Daratumumab and hyaluronidase (Darzalex Faspro^TM^) (given subcutaneously), the latter containing the same combined human mAb with a recombinant human enzyme called hyaluronidase, which enhances the absorption of injectables, allows faster infusions, and a lower rate of reactions related to infusions [29]. Both Darzalex^TM^ and Darzalex Faspro^TM^ target CD38. Approved by the FDA in 2005, human hyaluronidase injections alter the permeability of human tissue, and they are used as an adjuvant to improve the characteristics of injectables [30]. Other examples of mAb modification include Rituximab and hyaluronidase (Rituxan Hycela^TM^), approved in 2017, also given subcutaneously. However, it was first approved back in 1997 by the trade name Rituxan^TM^, being administered intravenously [31]. Trastuzumab and hyaluronidase (Herceptin Hylecta^TM^) [32] and Pertuzumab, trastuzumab, and hyaluronidase (Phesgo^TM^) [30] underwent the same modification with the addition of hyaluronidase, both being administered subcutaneously and both for breast cancer. Margenza™ is directed at the same target, HER2, for breast cancer [33], and all breast cancer biologicals currently on the market were approved between 2019 and 2020.

Lartruvo^TM^ was the only drug approved for soft tissue sarcoma during the period of interest [34]. Tecentriq^TM^, Bavencio^TM^ and Imfinzi^TM^ have the same target (PD-L1), and all three are biologicals that can be used to treat the highest number of different types of cancer [35,36,37]. Portrazza^TM^ targets EGFR, and Rybrevant^TM^ has the same target plus the MET proto-oncogene. Therefore, Rybrevant^TM^ is the only bispecific mAb for cancer approved to date [38,39]. Another breakthrough in the period 2015–2021 was Poteligeo^TM^, a first-in-class biopharmaceutical that targets the CC chemokine receptor 4 (CCR4) [40]. In this period, we found four biologicals approved for multiple myeloma, but one of them (Empliciti^TM^) has a distinct mechanism of action in that it binds to the cell surface receptor signaling lymphocytic activation molecule F7 (SLAMF7), whereas Darzalex^TM^, Darzalex Faspro^TM^ and Sarclisa^TM^ target CD38 [27,28,29]. Table 2 lists all the mAbs for cancer approved from 2015 to 2021 and detailed information for each one.

**Table 2:** Monoclonal antibodies for cancer approved by the Food and Drug Administration from 2015 to 2021.

Active Ingredient and Trade Name	mAb Class	Targets	Original Approval Date	Manufacturer	Therapeutic Indication
Empliciti^TM^ (Elotuzumab) [16,41]	Humanized	SLAMF7	2015	Bristol–Myers Squibb Company	Multiple myeloma
Portrazza^TM^ (Necitumumab) [16,38]	Human	EGFR	2015	Eli Lilly and Comp.	Squamous non-small cell lung cancer
Darzalex^TM^ (Daratumumab) [16,27]	Human	CD-38	2015	Janssen Biotech, Inc.	Multiple myeloma
Unituxin^TM^ (Dinutuximab) [16,42]	Chimeric	GD-2	2015	United Therapeutic Corporation	High-risk neuroblastoma
Tecentriq^TM^ (Atezolizumab) [17,36,43]	Humanized	PD-L1	2016	Genentech, Inc.	HCC, SCLC, TNBC, UC, NSCLC, and melanoma
Lartruvo^TM^ (Olaratumab) [34,43]	Human	PDGFR-α	2016	Eli Lilly and Comp.	Soft tissue sarcoma
Bavencio^TM^ (Avelumab) [37,44]	Human	PD-L1	2017	EMD Serono, Inc.	MCC, UC and RCC
Imfinzi^TM^ (Durvalumab) [35,44]	Human	PD-L1	2017	AstraZeneca UK Ltd.	UC, Stage III NSCLC and ES-SCLC
Rituxan Hycela^TM^ (Rituximab and hyaluronidase) [31,44]	Chimeric	CD-20	2017	Genentech, Inc.	DLBCL, CLL and follicular lymphoma
Libtayo™ (Cemiplimab) [19,45,46]	Human	PD-1	2018	Regeneron Pharmaceuticals, Inc.	CSCC, BCC, laBCC, mBCC and NSCLC
Poteligeo^TM^ (Mogamulizumab) [19,47]	Humanized	CCR-44	2018	Kyowa Kirin, Inc.	Mycosis fungoides or Sézary syndrome
Herceptin Hylecta^TM^ (Trastuzumab and hyaluronidase) [48,49]	Humanized	HER-2	2019	Genentech, Inc.	Breast cancer
Darzalex Faspro^TM^ (Daratumumab and hyaluronidase) [27,50]	Human	CD-38	2020	Janssen Biotech, Inc.	Multiple myeloma
Phesgo^TM^ (Pertuzumab, Trastuzumab and hyaluronidase) [30,50]	Humanized	HER-2	2020	Genentech, Inc.	Early or metastatic breast cancer
Monjuvi™ (Tafasitamab) [21,51]	Humanized	CD-19	2020	MorphoSys US Inc.	DLBCL
Danyelza™ (Naxitamab) [50,52]	Humanized	GD-2	2020	Y-mABs Therapeutics, Inc.	Neuroblastoma
Sarclisa^TM^ (Isatuximab) [28,50]	Chimeric	CD-38	2020	Sanofi-Aventis	Multiple myeloma
Margenza^TM^ (Margetuximab) [21,33]	Chimeric	HER-2	2020	MacroGenics Inc.	Metastatic breast cancer
Rybrevant^TM^ (Amivantamab) [2,39]	Human mAb	EGFR and MET	2021	Janssen Biotech, Inc.	NSCLC
Jemperli™ (Dostarlimab) [2,53]	Humanized	PD-1	2021	GlaxoSmithKline LLC	Endometrial cancer

CD—Cluster of Differentiation; PD-L1—Programmed Death Ligand 1; PDGFR-α—Platelet-Derived Growth Factor Receptor Alpha; EGFR—Epidermal Growth Factor Receptor; CCR—C-C Chemokine Receptor; SLAMF7—Signaling Lymphocytic Activation Molecule Family 7; GD—Glycolipid Disialoganglioside; HCC: Heptatocellular Carcinoma; TNBC: Triple-Negative Breast Cancer; SCLC: Small Cell Lung Cancer; NSCLC: Non-Small Cell Lung Cancer; BCC: Basal Cell Carcinoma; mBCC: Metastatic Basal Cell Carcinoma; CSCC: Cutaneous Squamous Cell Carcinoma; ES-SCLC: Extensive-Stage Small Cell Lung Cancer; RCC: Renal Cell Carcinoma; UC: Urothelial Carcinoma; MCC: Metastatic Merkel Cell Reactions Carcinoma; MET: a Proto-Oncogene; DLBCL: Diffuse Large B-Cell Lymphoma; and CLL: Chronic Lymphocytic Leukemia.

An important aspect of mAbs is their effectiveness compared to conventional treatments. For example, one of the main efficacy measures is overall survival (OS), and patients treated with Durvalumab, which is indicated for stage III non-small cell lung cancer, showed a higher OS than those receiving only chemotherapy (study also found in clinicaltrials.gov by the NCT03043872 trial number): median OS for Durvalumab + chemotherapy was 13 months while chemotherapy alone was 10 months [54].

#### 7.2.2. Antibody–Drug Conjugates

Enfortumab Vedotin Padcev^TM^ is the first biological to target the protein Nectin-4 [55]. Tisotumab Vedotin Tivdak^TM^ is a Biological specific for tissue factor (TF-011) and Polatuzumab Vedotin Polivy^TM^, an antibody whose target is the CD79b (a component of the B cell receptor). These three ADCs, which have different targets but the same suffix Vedotin, carry the same drug, namely monomethyl auristatin E (MMAE) [56,57,58]. MMAE is released into the cell after binding to the target, with subsequent induction of cell apoptosis by the drug, which also inhibits mitosis. These drugs also have different types of linkers. For example, the linker in Padcev^TM^ is the protease-cleavable maleimidocaproyl valine-citrulline [55], while Tisotumab Vedotin has a Valine citrulline linker, which is also protease-cleavable [57]. It is interesting how these ADCs carrying MMAE have such unique targets, a feature not seen among mAbs.

Fam-Trastuzumab deruxtecan Enhertu™ targets human epidermal growth factor receptor 2 (HER2) for the treatment of gastric cancer, breast cancer and gastroesophageal junction adenocarcinoma. Its ligand is a topoisomerase inhibitor, which blocks DNA replication [32]. Sacituzumab govitecan Trodelvy™, indicated to treat solid tumors, has the hydrolysis-cleavable CL2A as the linker, and it also carries a topoisomerase inhibitor [59]. Loncastuximab tesirine Zynlonta^TM^ includes an antibody against CD19. This antibody carries the antitumor drug pyrrolobenzodiazepine, and its linker is protease-cleavable [60].

Besponsa^TM^ has a linker that can be cleaved by acid [61]. Enhertu^TM^ has a protease-cleavable tetrapepitide linker [32,49]. Trodelvy™ has the hydrolysis-cleavable CL2A as linker [59]. The linker present in Zynlonta^TM^ is also protease-cleavable [60] while that of Blenrep^TM^ is maleimidocaproyl [62].

Besponsa^TM^ and Lumoxiti™ target CD22, but they are indicated for different types of cancer [11,61]. They carry distinct drugs/toxins, Besponsa^TM^ carrying Calich-DMH, an antitumor antibiotic produced by a bacterium, and Lumoxiti^TM^ being conjugated to a fragment of *Pseudomonas exotoxin,* also found as PE38. When internalized, PE38 stimulates apoptosis and the inhibition of protein synthesis. Table 3 shows the ADCs and information related to approval date, targets, manufacturer, name, and origin. Of note, all ADCs approved are indicated to treat cancers.

**Table 3:** Antibody–Drug Conjugates approved by the Food and Drug Administration from 2015 to 2021.

Active Ingredient and Trade Name	Antibody Class	Targets	Original Approval Date	Manufacturer	Therapeutic Indication
Besponsa™ (Inotuzumab ozogamicin) [44,61]	Humanized	CD22	2017	Wyeth Pharmaceuticals LLC	B-cell precursor ALL
Lumoxiti^TM^ (Moxetumomab pasudotox) [11,45]	Murine	CD22	2018	Innate Pharma, Inc.	Hairy cell leukemia
Padcev^TM^ (Enfortumab Vedotin) [55,56]	Human	Nectin-4	2019	Astellas PharmaUS, Inc.	Metastatic urothelial cancer
Polivy™ (Polatuzumab Vedotin) [48,58]	Humanized	CD79b	2019	Genentech, Inc.	Diffuse large B-cell lymphoma
Enhertu^TM^ (Fam-Trastuzumab deruxtecan) [32,48]	Humanized	HER-2	2019	Daiichi Sankyo, Inc.	Breast cancer and gastric or gastroesophageal junction adenocarcinoma
Trodelvy™ (Sacituzumab govitecan) [21,59]	Humanized	Glycoprotein Trop-2	2020	Gilead Sciences, Inc.	mTNBC
Blenrep^TM^ (Belantamab Mafodotin) [50,62]	Humanized	BCMA	2020	GlaxoSmithKline Intellectual Property Development Ltd.	Multiple myeloma
Zynlonta™ (Loncastuximab tesirine) [2,60]	Chimeric	CD19	2021	ADC Therapeutics SA	Diffuse large B-cell lymphoma
Tivdak™ (Tisotumab Vedotin) [2,57]	Human	Tissue factor (TF-011)	2021	Seagen Inc.	Metastatic cervical cancer

TF—Tissue Factor; CD—Cluster of Differentiation; HER—Human Epidermal Growth Factor Receptor; BCMA—B-cell Maturation Antigen; ALL: Acute Lymphocytic Leukemia; mTNBC: Metastatic Triple-Negative Breast Cancer.

In 2015 and 2016, no ADCs were approved, while 2017 and 2018 registered the lowest number of authorizations of these drugs in the period of interest. In 2019, the highest number of approvals for ADCs were for the treatment of three types of cancer. In this regard, Padcev^TM^ was authorized for the treatment of metastatic urothelial cancer [55], Polivy^TM^ for diffuse large B-cell lymphoma [58], and Enhertu^TM^ for breast cancer [32]. Then, the following two years registered two approvals each year. Thus, in 2020, Blenrep^TM^ received the green light for the treatment of multiple myeloma [62] and Trodelvy^TM^ for metastatic triple-negative breast cancer [59]. In the following year, Zynlonta^TM^, another drug for the treatment of large B-cell Lymphoma [60], was approved, as was Tivdak^TM^ for metastatic cervical cancer [57].

As seen earlier in this review, ADCs can carry a variety of antitumor components. Table 4 shows MMAE and other drugs that were found for the approved ADCs. 

**Table 4:** Antibody–Drug Conjugates and their respective drugs.

Drug/Toxin/Chemotherapy	Antibody–Drug Conjugate
Monomethyl auristatin E (MMAE) [56,57,58]	Enfortumab Vedotin Tisotumab Vedotin Polatuzumab Vedotin
Calich-DMH [61]	Inotuzumab ozogamicin
Topoisomerase inhibitor [32,59]	Fam-Trastuzumab deruxtecan Sacituzumab govitecan
Pyrrolobenzodiazepine [60]	Loncastuximab tesirine
fragment of *Pseudomonas exotoxin* [11]	Moxetumomab pasudotox
Maleimidocaproyl monomethyl auristatin F (mcMMAF) [62]	Belantamab mafodotin

Regarding the efficacy of ADCs, they show excellent performance with respect to OS. For example, one of the ADCs approved (Enfortumab Vedotin for advanced urothelial carcinoma) had a superior median OS of 12.88 months when compared to chemotherapy alone, with an OS of 8.97 months [56] (study also found in clinicaltrials.gov by NCT03474107).

Of the 32 biopharmaceuticals approved for the treatment of cancer in the period of interest, 29 are in the classes listed in Table 2 and Table 3, the rest falling into the categories of enzymes and fusion proteins (Table 5). Of note during the period was the approval of a unique treatment for Blastic Plasmacytoid Dendritic Cell Neoplasm (BPDCN) (Tagraxofusp Elzonris^TM^), a disease for which no standard treatment had been available previously. The literature reports a better response to this drug in treatment-naïve patients than in those who had already been treated with other therapies for BPDCN, and a 90% overall response rate (ORR) in 70-year-old patients [63,64]. To date, the literature also shows limited data regarding this new treatment, and further evaluation is needed.

**Table 5:** Enzymes and proteins for cancer approved by the Food and Drug Administration from 2015 to 2021.

Active Ingredient and Trade Name	Biological Class	Target/Mechanism of Action	Original Approval Date	Manufacturer	Therapeutic Indication
Asparlas™ (Calaspargase pegol) [19,65]	Enzyme	Conversion of amino acids	2018	Servier Pharmaceuticals	ALL
Elzonris™ (Tagraxofusp) [19,64]	Fusion protein	CD-123	2018	Stemline Therapeutics Inc.	BPDCN
Rylaze™ (Asparaginase erwinia chrysanthemi (recombinant)) [2,66]	Enzyme	Conversion of amino acids	2021	Jazz Pharmaceuticals Ireland Limited	ALL and LBL

CD—Cluster of Differentiation; ALL: Acute Lymphoblastic Leukemia; LBL: Lymphoblastic Leukemia; BPDCN: Blastic Plasmocytoid Dendritic Cell Neoplasm.

## 8. Autoimmune Diseases

The biologics for autoimmune diseases (psoriasis, plaque psoriasis, psoriatic arthritis, multiple sclerosis, myasthenia gravis, lupus erythematosus, rheumatoid arthritis, ankylosing spondylitis, Crohn’s disease, ulcerative colitis, and neuromyelitis optic spectrum disorder) in the period of interest included 13 biologics, 12 of which were mAbs, and a new class of biological, namely an antibody fragment (Efgartigimod alfa Vyvgart^TM^), which is detailed in Table 6. 

**Table 6:** Biopharmaceuticals for autoimmune diseases approved by the Food and Drug Administration from 2015 to 2021.

Active Ingredient and Trade Name	mAb Class	Targets/Mechanism of Action	Original Approval Date	Manufacturer	Therapeutic Indication
Cosentyx™ (Secukinumab) [16,67]	Human	IL-17A inhibitor	2015	Novartis Pharmaceuticals	Plaque psoriasis, Psa, and AS
Zinbryta™ (daclizumab) [43,68]	Humanized	IL-2R inhibitor	2016	Biogen Inc	Multiple sclerosis
Taltz™ (ixekizumab) [43,69]	Humanized	IL-17A inhibitor	2016	Eli Lilly and Company	Plaque psoriasis and Psa
Tremfya™ (guselkumab) [18,70]	Human	IL-23 and IL-17A inhibitor	2017	Janssen Biotech, Inc	Plaque psoriasis
Ocrevus™ (Ocrezilumab) [44,71]	Humanized	Anti-CD-20	2017	Genentech, Inc	Multiple sclerosis
Kevzara™ (sarilumab) [44,72]	Human	IL-6 inhibitor	2017	Sanofi-Aventis U.S LLC	Rheumatoid arthritis
Siliq™ (brodalumab) [44,73]	Human	IL-17A, IL-17F, and other IL-17 isoform inhibitors	2017	Valeant Pharmaceuticals Luxembourg S.à.r.l	Plaque psoriasis
Ilumya™ (tildrakizumab) [45,74]	Humanized	IL 23p19	2018	Sun Pharma Global FZE	Plaque psoriasis
Skyrizi™ (risankizumab) [48,75]	Humanized	IL-23p19 inhibitor	2019	AbbVie Inc.	Plaque psoriasis and Psa
Uplizna™ (inebilizumab) [50,76]	Humanized	Depletes CD-19	2020	Horizon Therapeutics Ireland DAC	NMOSD
Enspryng™ (satralizumab) [21,77,78]	Humanized	Anti-IL -6R	2020	Genentech, Inc.	NMOSD
Saphnelo™ (anifrolumab) [2,79]	Human	Blocks the action of type 1 interferon receptor	2021	AstraZeneca AB	Lupus erythematosus
Vyvgart™ (efgartigimod alfa) [2,80]	Human monoclonal ARGX-113 fc fragment	Neonatal Fc receptor antagonist	2021	Argenx BV	Generalized myasthenia gravis

IL—Interleukin; CD—Cluster of Differentiation; Psa: Psoriatic Arthritis; NMOSD: Neuromyelitis Optica Spectrum Disorder.

### Mechanism of Action and Therapeutic Indications for Autoimmune Diseases

Of the approvals of autoimmune biopharmaceuticals from 2015 to 2021, six are indicated for psoriasis, plaque psoriasis, and psoriatic arthritis. Brodalumab Siliq™ is indicated for moderate to severe plaque psoriasis [73]. While this drug acts by antagonizing the IL-17A Receptor, Cosentyx™ and Taltz™ antagonize the pro-inflammatory cytokine IL-17A, which plays a role in psoriasis and Psa [67,69]. Guselkumab Tremfya™, used for the treatment of psoriasis and Psa, is an antibody that blocks the activity of two interleukins (IL-23, IL-17A) that are overexpressed in these diseases [71]. Tildrakizumab Ilumya™ is an IgG1 antibody that selectively binds to interleukin-23-p19 (IL-23A p19) [74] and, through the same mechanism, Risankizumab Syrizi™ also binds to the same p19 subunit of this interleukin. In some countries, there are trials underway to evaluate Risankizumab for the treatment of Crohn’s disease and ulcerative colitis [75,81,82].

Saphnelo™, which has a unique mechanism of action, was the only biologic found between 2015 and 2021 for the treatment of Lupus Erythematosus [79]. Sarilumab Kevzara™ is an IgG1 monoclonal antibody, the only one for rheumatoid arthritis approved within the period of interest [72]. In this regard, two biologicals with indications for multiple sclerosis were found. Zinbryta™, which is indicated only when there has been an inadequate patient response to two other DMTs (disesase-modifying therapies) [83], and Ocrelizumab Ocrevus™, were approved in 2016 and 2017, respectively [71].

Neuromyelitis optic spectrum disorder is a demyelinating disease of the CNS, optic nerve, and spinal cord. Patients with this disorder show levels of interleukin-6 (IL-6) in the cerebrospinal fluid that are above normal [78]. Enspryng™ binds to the interleukin 6 receptor (IL-6R), preventing IL-6 from binding to its receptor [77]. The other drug used to treat this condition, namely Uplizna™, binds to CD-19 [76]. mAbs commonly target interleukin receptors.

## 9. Other Therapeutic Indications

In the period of interest, some therapeutic indications appear only once among FDA approvals, while others appear between two to four times. Of a total of four FDA-approved mAbs for the treatment of migraine, three are humanized mAbs and only one is fully human (Table 7). The humanized ones, Vyepti™, Emgality™ and Ajovy™, have the same mechanism of action. In this regard, they bind to CGRP, a potent vasodilator, preventing it from adhering to the receptor [84,85,86]. In contrast, the fully human antibody Aimovig™ binds to CGRPR, preventing the molecule from binding to the receptor [87].

**Table 7:** Monoclonal antibodies for migraine approved by the Food and Drug Administration from 2015 to 2021.

Active Ingredient and Trade Name	mAb Class	Target/Mechanism of Action	Original Approval Date	Manufacturer
Emgality™ (Galcanezumab) [45,87]	Humanized	CGRP antagonist	2018	Eli Lilly and Company
Ajovy™ (Fremanezumab) [45,86]	Humanized	CGRP antagonist	2018	Teva Branded Pharmaceutical Products R&D, Inc.
Aimovig™ (Erenumab) [45,87]	Human	CGRPR antagonist	2018	Amgen, Inc.
Vyepti™ (Eptinezumab) [21,84]	Humanized	CGRP antagonist	2020	Lundbeck Seattle Pharmaceuticals, Inc.

CGRP: Calcitonin Gene-Related Peptide; CGRPR: Calcitonin Gene-Related Peptide Receptor.

For asthma and severe asthma (Table 8), two fully human antibodies were approved from 2015 to 2021. Dupixent™ is an antibody directed against the α subunit of the interleukin 4 receptor (IL-4R-α) [88], and the Tezsipre™ blocks thymic stromal lymphopoietin (TSLP), which plays a key role in asthma [89,90]. Furthermore, in the same period, three humanized antibodies received the green light. In this regard, Nucala™ and Cinqair™ are mAbs against IL-5 [91,92], while Fasenra™ acts by binding to the α subunit of the receptor of IL-5 (IL-5R-α) [90,93].

**Table 8:** Monoclonal antibodies for asthma and severe asthma approved by the Food and Drug Administration from 2015 to 2021.

Active Ingredient and Trade Name	mAb Class	Target/Mechanism of Action	Original Approval Date	Manufacturer
Nucala™ (Mepolizumab) [16,91]	Humanized	IL-5	2015	GlaxoSmithKline LLC
Cinqair™ (Reslizumab) [43,92]	Humanized	IL-5	2016	Teva Respiratory LLC
Fasenra™ (Benralizumab) [44,93]	Humanized	IL-5R-α	2017	AstraZeneca AB
Dupixent™ (Dupilumab) [44,88]	Human	IL-4R-α	2017	Regeneron Pharmaceuticals, Inc.
Tezsipire™ (Tezepelumab) [2,90]	Human	Blocks TSLP	2021	AstraZeneca AB

IL: Interleukin; IL-R-α: Interleukin Receptor α; TSLP: Thymic Stromal Lymphopoietin.

One of the key aspects of biologicals is their potential for the treatment of rare diseases, such as type 2 neuronal ceroid lipofuscinosis, which causes symptoms ranging from seizures and loss of motor coordination to vision failure. The diagnosis of this condition can be delayed due to the similarity of symptoms with other diseases. This disease causes blindness in children, a patient cohort that can make it difficult to conduct clinical trials due to a smaller population for initial studies [94]. For this disease, also known as Batten’s disease, only one biological, an enzyme (Cerliponase alfa Brineura™), was approved during the period of interest [95].

The 2021 Biological of the year is Aducanumab Aduhelm™, which was authorized in the midst of the COVID-19 pandemic through an accelerated FDA approval, despite controversy regarding phase three studies, which showed that the drug is associated with a decrease in beta-amyloid plaques. However, these studies have not proved satisfactorily that the drug delays cognitive and functional decline in patients with AD. New FDA submissions of biologicals to treat AD will soon emerge, such as Lecanemab and Donanemab, which are currently in the testing phase. However, these two drugs still need further supporting clinical evidence to compete with Aducanumab and enter the market [23,96,97]. As mentioned earlier, the EMA withdrew marketing authorization for Aducanumab.

Between 2015 and 2021, biologicals were also approved for the treatment of *Bacillus anthracis* (Obiltoxaximab Anthim™), Pseudomembranous Colitis (Bezlotoxumab Zinpala™), Hemophilia A (Emicizumab Hemlibra™), Sly Syndrome (Vestronidase alfa Mepsevii™), X-linked hypophosphatemic rickets (Burosumab Crysvita™), neurotrophic keratitis (Cenegermin Oxervate™), drug-resistant HIV-1 (Ibalizumab Trogarzo™), phenylketonuria (Pegvaliase Palynziq™), temporary smoothing of fine lines (Prabotulinumtoxin Jeuveau™), growth deficiency (Somapacitan Sogroya™, Lonapegsomatropin Skytrofa™), and Ebola virus (Atoltivimab, Maftivimab, and Odesivimab Inmazeb™ and Ansuvimab Ebanga™), among others (Table 9). Within the context of ‘biological treatment’, it can be concluded that one of the perspectives is to increasingly promote options for the treatment of patients with chronic diseases, including rare conditions [98]

**Table 9:** Other biologicals approved by the Food and Drug Administration from 2015 to 2021.

Active Ingredient and Trade Name	Biological Class	Target/Mechanism of Action	Original Approval Date	Manufacturer	Therapeutic Indication
Natpara^TM^ (parathyroid hormone) [16,99]	Hormone	Supplies parathyroid hormone	2015	NPS Pharmaceutical Inc.	Control of hypocalcemia in hypoparathyroidism
Praluent^TM^ (Alirocumab) [16,100]	Human mAb	Inhibits PCSK9 from binding to LDLR	2015	Regeneron Pharmaceutical Inc.	High cholesterol
Repatha^TM^ (Evolocumab) [16,101]	Human mAb	Inhibits PCSK9 from binding to LDLR	2015	Amgen Inc.	High cholesterol
Tresiba^TM^ (insulin degludec) [16]	Human insulin	Stimulates peripheral glucose intake	2015	Novo Nordisk Inc.	Improves glucose control in diabetes mellitus
Praxbind^TM^ (Idarucizumab) [16,102]	Humanized mAb fragment	Binds to dabigatran and neutralizes its anticoagulant effects	2015	Boehringer Igelheim Pharm.	Patients treated with Pradaxa^TM^ when the reversal of anticoagulant effect is needed
Strensiq^TM^ (Asfotase alfa) [16,103]	Enzyme	Replacement of TNSALP upon asfotase alfa	2015	Alexion Pharmaceuticals, Inc.	Hypophosphatasia
Kanuma^TM^ (Sebelipase alfa) [16,104]	Enzyme	Catalyzes the lysosomal hydrolysis of cholesteryl and triglycerides	2015	Alexion Pharmaceuticals, Inc.	Lysosomal Acid Lipase deficiency
Anthim^TM^ (Obiltoxaximab) [43,105]	Humanized mAb	Acts against the protective antigen of *Bacillus anthracis*	2016	Elusys Therapeutics, Inc.	Anthrax
Zinplava^TM^ (Bezlotoxumab) [43,106]	Human mAb	Binds to *Clostridium difficile* toxin B	2016	Merck Sharp & Dohme Corp.	*Clostridium difficile* infection
Hemlibra^TM^ (Emicizumab) [44,107,108]	Humanized mAb	Factor X and factor IXa	2017	Genentech, Inc.	Hemophilia A
Brineura^TM^ (Cerliponase alfa) [44,95]	Enzyme	Hydrolytic lysosomal N-terminal tripeptidyl peptidase	2017	BioMarin Pharmaceutical Inc.	Neuronal CLN2
Mepsevii^TM^ (Vestronidase alfa) [44,109]	Enzyme	Degrades GAG	2017	Ultragenyx Pharmaceutical Inc.	MPS VII, Sly syndrome
Crysvita^TM^ (Burosumab) [45,110]	Human mAb	Inhibits FGF23	2018	Kyowa Kirin, Inc.	X-linked dominant hypophosphatemic rickets
Oxervate^TM^ (Cenegermin) [45,111]	Protein	Important nerve growth factor for the survival of neurons	2018	Dompé farmaceutici S.p.A.	Neurotrophic keratitis
Revcovi^TM^ (Elapegademase) [45,112]	Pegylated enzyme	External supply of ADA enzyme	2018	Chiesi USA, Inc.	ADA-SCID
Gamifant^TM^ (Emapalumab) [45,113]	Human mAb	Neutralizes Interferon gamma (IFNγ)	2018	Swedish Orphan Biovitrum AB (publ)	Hemophagocytic lymphohistiocystosis
Trogarzo^TM^ (Ibalizumab) [45,114]	Humanized mAb	Viral entry inhibitor, blocking viral entry into CD4 cells	2018	Theratechnologies Inc.	Multidrug-resistant HIV-1
Takhzyro^TM^ (Lanadelumab) [45,115]	Human mAb	Binds to plasma kallikrein	2018	Takeda Pharmaceuticals U.S.A., Inc.	Hereditary angioedema attacks
Palynziq^TM^ (Pegvaliase) [45,116]	Pegylated enzyme	Conversion of amino acid into ammonia and trans-cinnamic acid	2018	BioMarin Pharmaceutical Inc.	Phenylketonuria
Ultomiris^TM^ (Ravulizumab) [45,117]	Humanized mAb	Inhibits cleavage of C5 to C5a and C5b	2018	Alexion Pharmaceuticals, Inc.	PNH and atypical hemolytic uremic syndrome
Beovu^TM^ (Brolucizumab) [48,118]	Humanized mAb	Inhibits VEGF	2019	Novartis Pharmaceuticals Corp.	Wet age-related macular degeneration
Cablivi^TM^ (Caplacizumab) [48,119]	Humanized mAb	Inhibits interactivity vWF-platelets, reducing platelet adhesion	2019	Ablynx NV	aTTP
Adakveo^TM^ (Crizanlizumab) [48,120]	Humanized mAb	Binds to P-Selectin	2019	Novartis Pharmaceuticals Corp.	Prevention of VOC in Sickle cell disease
Evenity^TM^ (Romosozumab) [48,121]	Humanized mAb	Inhibits sclerostin, increasing bone formation	2019	Amgen, Inc.	Osteoporosis in postmenopausal women
Reblozyl^TM^ (Luspatercept) [48,122]	Fusion protein	Diminishes Smad-2/3 signaling pathway	2019	Celgene Corp., a Bristol-Myers Squibb Company	Anemia in β-thalassemia and myelodysplastic sydromes
Jeuveau^TM^ (Prabotulinumtoxin) [48,123]	Neurotoxin protein	Inhibits the release of acetylcholine in nerve terminals	2019	Evolus Inc.	Temporary improvement of grabellar and frown lines
Ebanga^TM^ (Ansuvimab) [50,124]	Human mAb	Inhibits Ebola virus from binding to NPC1 receptor	2020	Ridgeback Biotherapeutics	Ebola virus
Inmazeb^TM^ (Atoltivimab, Aaftivimab, Odesivimab) [50,125]	Human mAbs	Inhibits *Zaire Ebola virus*	2020	Regeneron Biopharmaceuticals, Inc.	Ebola virus
Sogroya^TM^ (Somapacitan) [50,126]	Protein	Replacement of endogenous growth hormone	2020	Novo Nordisk Inc.	Growth hormone deficiency
Tepezza^TM^ (Teprotumumab) [50,127]	Human mAb	Inhibits Insulin-like growth factor-1 receptor	2020	Horizon Therapeutics Ireland DAC	Thyroid eye disease
Aduhelm™ (Aducanumab) [2,96]	Human mAb	Anti-amyloid beta	2021	Biogen, Inc.	Alzheimer’s Disease
Adbry^TM^ (Tralokinumab) [99,128]	Human mAb	Binds to IL-13 and inhibits it from binding to IL-13R α1 and α2 subunits	2021	LEO Pharma A/S	Atopic dermatitis
Besremi^TM^ (Ropeginterferon alfa) [2,129]	Pegylated enzyme	Type I interferon, it binds to IFANR	2021	PharmaEssentia Corporation	Polycythemia vera
Evkeeva^TM^ (Evinacumab) [99,130]	Human mAb	Inhibits ANGPTL3	2021	Regeneron Pharmaceuticals, Inc.	Homozygous familial hypercholesterolemia
Nexviazyme^TM^ (Avalglucosidade alfa) [99,131]	Enzyme	External source of GAA	2021	Genzyme Corporation	Glycogen storage disease type 2 (Pompe disease)
Skytrofa^TM^ (Lonapegsomatropin) [99,132]	Pegylated hormone	Binds to the GH receptor	2021	Ascendis Pharma Endocrinology Divison A/S	Growth hormone deficiency

GAG: Glucuronate-Containing Glycosaminoglycan; CD: Cluster of Differentiation; IL-R: Interleukin Receptor; LDLR: Low Density Lipoprotein Receptor; TNSALP: Tissue-Nonspecific Alkaline Phosphatase; Neuronal CLN2: Ceroid Lipofuscinosis Type 2; MPS: Mucopolysaccharidosis; ADA-SCID: Adenosine Deaminase Severe Combined Immunodeficiency; VOC: Vaso-Occlusive Crisis; aTTP: Acquired Thrombotic Thrombocytopenic Purpura; NPC1: Niemann-Pick C1 Receptor; FGF: Fibroblast Growth Factor; PNH: Paroxysmal Nocturnal Hemoglobinuria; VEGF: Vascular Endothelial Growth Factor; IFANR: Interferon α Receptor; ANGPTL3: Angiopoietin-like 3; GAA: Acid α-Glucosidase; GH: Growth Hormone; PCSK9: Proprotein Convertase Subtilisin Kexin Type 9.

## 10. Discussion

The period 2015 to 2021 witnessed a growth in FDA approval of biologicals in general, with mAbs being the class with the greatest presence. During this period, the number of authorizations of biopharmaceuticals remained in the double figures, except in 2016, when only seven were given the green light. The years 2020 and 2021 did not show considerable variation, with one less biological being approved in 2021 than in 2020, while 2018 was the year with the highest number of approvals. Of note, even in the midst of the COVID-19 pandemic, the potential for these therapies to receive approval remained steady.

From the perspective of the origin of the biologicals, more humanized antibodies were approved than fully human, followed by chimerics, and only one biopharmaceutical from the murine class—an ADC. The authorization of only one murine mAb could be because these biologicals already posed a risk of immunogenicity to patients decades ago [5].

Among the therapeutic indications for which biopharmaceuticals were authorized in the period of interest, some appear to be more common targets. In this regard, the most common therapeutic indication was cancer. This can be explained by the fact that cancer is one of the main causes of death worldwide and biopharmaceuticals can be conjugated to drugs, thereby targeting cancer cells more selectively, and, importantly, decreasing toxicity. The latter aspect is highly relevant as most antitumor treatments are toxic. Of the 90 biologicals approved, 34% (32) (half of these being mAbs) target different types of cancer. Of note, many of the mAbs and protein classes approved were indicated for cancer. However, other therapeutic indications were also found for these drugs. In contrast, all the ADCs approved during the period of interest were for the treatment of cancer. In this regard, three ADCs were authorized in 2019, two in 2020, and two in 2021, in contrast to only one approval in 2017 and one in 2018. Although the same cytotoxic payload was repeated in some ADCs, all the antibody classes were present within the ADCs, which is highly significant. Other classes of drugs showed a much lower rate of approval. However, important ones, such as the fusion protein Elzonris^TM^, the first approval for BPDCN [12], were a real breakthrough.

Biological medicines show high selectivity and high versatility and are therefore valuable. Their versatility is reflected in indications that range from the treatment of chronic or rare diseases to more aesthetic purposes such as the treatment of frown lines. These drugs offer great potential to be exploited for other therapeutic indications beyond what they were initially authorized for. In this regard, they offer a solid starting point from which to explore their capacity in clinical trials. For example, over the years, new applications have been discovered for Adalimumab Humira^TM^, and today this drug has more than ten therapeutic indications listed in the directions of use [133]. Daratumumab Darzalex^TM^ is also undergoing evaluation for other types of cancer, including refractory or relapsed non-Hodgkin’s Lymphoma [27]. mAbs can also be conjugated to toxins or drugs without compromising healthy tissues around the target fragment or at least minimizing effects in other tissues [134]. Apart from mAbs, we found that potential to be further evaluated for other therapeutic indications also in the antibody namely Efgartigimod Vyvgart^TM^ in the future [80]. 

Regarding Aduhelm^TM^, although its average annual price is being criticized, the next drug approved for AD could have excellent financial potential given that the last innovation in the treatment of this condition approved was in 2003. In addition, given the average price of mAbs and the challenge to find a therapeutic innovation for this neurodegenerative disease, any new therapy would undoubtedly carry with it a significant cost. As seen in this work, the authorizations of biologicals for certain therapeutic indications grew considerably in the period 2015–2021, with mAbs as the category that received the most approvals by the FDA. 

Between 2015 and 2021, in addition to the increase in the number of drug approvals, several breakthroughs and innovations took place, such as Aducanumab Aduhelm^TM,^ although still controversial, and also Tagraxofusp Elzonris^TM^, which the FDA granted the status of Orphan Drug to treat rare diseases. In 2021, we witnessed the authorization of a different class of biological, Efgartigimod alfa Vyvgart^TM^, an antibody fragment that also has Orphan Drug status [80], and the bispecific antibody approved within the period of interest Hemlibra^TM^. Of note only two bispecific antibodies were approved in the period of interest Hemlibra^TM^ and Rybrevant^TM^.

Although 2021 was not the year with the highest number of biological drug authorizations, 14 did obtain the green light in the midst of the COVID-19 pandemic, a number that was still above the annual average over the period addressed in this review. 

## 11. Conclusions

In the period 2015–2021, cancer continued to be the main target, but there was increasing interest in discovering new ways and new targets, reflected, for example, by a new class of biological as a fragment of an antibody (Efgartigimod) [80], the first therapy targeting Nectin-4 (Enfortumab Vedotin) [55,56]. This is the first direct therapy to date for BPDCN and also the first treatment to target CD123 [63,64], while Aducanumab is the first drug for AD whose target is amyloid-beta. 

The pharmaceutical industry is becoming increasingly aware that living organisms are an excellent source of inspiration.

However, one of the great challenges for the development of biopharmaceuticals is the high technology required to produce these drugs, which makes them very expensive. We believe that, in the near future, this class of drugs will become increasingly accessible and new drugs will be developed. Moreover, more biosimilars will become accessible thanks to the development of new technologies that will impact production. These advancements will make these drugs increasingly more profitable and less expensive, which in turn will widen the accessibility of biological therapies, thereby expanding the therapeutic arsenal and transforming the management of diseases for which no treatment is available or diseases for which current treatments are not effective.

## Figures and Tables

**Table 1:** Nomenclature of mAb-based biologicals according to the origin of the antibody [6].

Stem of Biologicals	Substem B of Biologicals	Examples of Biologicals
Suffix -mab (monoclonal antibodies)	-u- (human)	Adalimumab
	-zu- (humanized)	Morgamulizumab
	-xi- (chimeric)	Dinutuximab
	-o- (murine)	Muronomab

## Data Availability

Not applicable.
